# 不同室间隔形态肺高血压患者的心室功能特点：CMR初步研究

**DOI:** 10.3779/j.issn.1009-3419.2018.05.07

**Published:** 2018-05-20

**Authors:** 丹 王, 璋 张, 帆 杨, 乐 张, 振文 杨, 雯 任, 铁链 于, 东 李

**Affiliations:** 1 300052 天津，天津医科大学总医院医学影像科 Department of Radiology, Tianjin Medical University General Hospital, Tianjin 300052, China; 2 300052 天津，天津医科大学总医院心血管内科 Department of Cardiovascular Disease, Tianjin Medical University General Hospital, Tianjin 300052, China

**Keywords:** 心脏磁共振, 室间隔, 形态, 肺高血压, 肺动脉, 心室功能, Cardiovascular magnetic resonance, Interventricular septum, Shape, Pulmonary hypertension, Pulmonary artery, Ventricular function

## Abstract

**背景与目的:**

通过心脏磁共振（cardiovascular magnetic resonance, CMR）分析室间隔（interventricular septum, IVS）有无形变的肺高血压（pulmonary hypertension, PH）患者心功能特点。

**方法:**

经右心导管确诊为PH并接受CMR患者36例。根据IVS形态，分为无形变组（10例）和有形变组（26例）；并与22例健康志愿者比较，参数如下：右心室（right ventricle, RV）和左心室（left ventricle, LV）舒张末期容积指数（end-diastolic volume index, EDVI）、收缩末期容积指数（end-systolic volume index, ESVI）、每搏输出量指数（stroke volume index, SVI）、心指数（cardiac index, CI）、射血分数（ejection fraction, EF）、心肌质量指数（myocardial mass index, MMI）。

**结果:**

ANOVA分析示，RVEDVI、RVESVI、RVSVI、RVCI、RVEF、RVMMI、LVEDVI、LVESVI、LVSVI及LVCI在三组间差别均有统计学意义。事后组间结果比较显示，PH患者IVS无形变组与对照组相比，RVSVI（*P*=0.017）、RVEF（*P* < 0.001）、LVEDVI（*P*=0.048）、LVSVI（*P*=0.015）均减低。IVS有形变组与IVS无形变组相比，RVEDVI（*P* < 0.001）、RVESVI（*P* < 0.001）、RVCI（*P*=0.002）、RVMMI（*P*=0.017）均升高；而RVEF（*P*=0.001）、LVEDVI（*P*=0.003）、LVSVI（*P* < 0.001）及LVCI（*P*=0.029）减低。IVS有形变组与对照组相比，RVEDVI（*P* < 0.001）、RVESVI（*P* < 0.001）、RVCI（*P*=0.004）、RVMMI（*P*=0.003）均升高；而RVEF（*P* < 0.001）、LVEDVI（*P* < 0.001）、LVESVI（*P* < 0.001）、LVSVI（*P* < 0.001）、LVCI（*P* < 0.001）均低于对照组。

**结论:**

不同IVS形态的PH患者，心室功能各有特点，IVS的形变在一定程度上能够反映PH患者心室功能的变化。

## Abstract (en)

**Background and objective:**

To study the characteristics of ventricular function in Pulmonary Hypertension (PH) Patients with different shape of Interventricular Septum (IVS) by cardiac magnetic resonance (CMR).

**Methods:**

36 PH patients diagnosed by right heart catheterization accepted CMR. According to the morphology of IVS, the patients were divided into two groups: the non-deformation group (10 patients) and the deformation group (26 patients). The ventricular function parameters were as follows: RV and LV end-diastolic volume index (EDVI), end-systolic volume index (ESVI), stroke volume index (SVI), cardiac index (CI), ejection fraction (EF), and myocardial mass index (MMI).

**Results:**

ANOVA analysis showed that the differences of RVEDVI, RVESVI, RVSVI, RVCI, RVEF, RVMMI, LVEDVI, LVESVI, LVSVI and LVCI were significant among the three groups. Compared with control group, RVSVI (*P*=0.017), RVEF (*P* < 0.001), LVEDVI (*P*=0.048) and LVSVI (*P*=0.015) decreased in IVS non-deformation group. Compared with IVS non-deformation group, RVEDVI (*P* < 0.001), RVESVI (*P* < 0.001), RVCI (*P*=0.002) and RVMMI (*P*=0.017) were increased in IVS deformation group; while RVEF (*P*=0.001), LVEDVI (*P*=0.003), LVSVI (*P* < 0.001) and LVCI (*P*=0.029) were decreased. Compared with the control group, RVEDVI (*P* < 0.001), RVESVI (*P* < 0.001), RVCI (*P*=0.004) and RVMMI (*P*=0.003) were increased in the IVS deformation group, while RVEF (*P* < 0.001), LVEDVI (*P* < 0.001), LVESVI (*P* < 0.001), LVSVI (*P* < 0.001), LVCI (*P* < 0.001) were decreased.

**Conclusion:**

Ventricular function is different in PH Patients with different IVS shape. The IVS shape can represent the changes of ventricular function in PH patients.

左心室（left ventricle, LV）和右心室（right ventricle, RV）共享室间隔（interventricular septum, IVS），在心动周期中，IVS位置形态是由LV和RV的压力差，即跨IVS压力梯度决定^[1]^。肺高血压（pulmonary hypertension, PH）是肺动脉压力持续高于正常的病理状态，PH压力超负荷可导致RV心肌壁肥厚和心腔扩大，甚至IVS发生形变^[2, 3]^。心脏磁共振（cardiac magnetic resonance, CMR）成像在评估心脏形态结构功能、量化心室容积、质量等方面具有独特优势^[4]^。本研究拟通过CMR成像来探讨IVS不同形态下PH患者RV和LV功能的特点。

## 1 材料和方法

### 1.1 研究对象

选取2014年10月-2017年2月在天津医科大学总医院经右心导管检查（right heart catheterization, RHC）确诊并接受CMR检查的PH患者36例。均排除了冠心病、心脏瓣膜病、慢性阻塞性肺疾病等其他心肺疾病，且无严重肾功能不全及MR检查禁忌症，能配合完成检查。另纳入健康志愿者22例作为正常对照组，其心率、血压均在正常范围，无心肺疾病、代谢综合征等病史并接受CMR检查。本研究经天津医科大学总医院伦理委员会批准，所有受检者对此项研究知情同意。

### 1.2 CMR检查设备与扫描方法

采用GE 3.0T Twin-speed Infinity with Excite Ⅱ超导型MR扫描仪（GE Healthcare, Milwaukee, WI, USA），8通道相控阵线圈，心电门控和呼吸门控进行呼气末屏气采集CMR图像。采用二维快速稳态进动采集序列（fast imaging employing steady-state acquisition, FIESTA）获得心脏短轴位和四腔心位图像。成像参数：TR/TE minfull/minfull，带宽125 kHz，翻转角45°，矩阵224×224，NEX 1，扫描层厚8 mm，层间距0 mm，FOV 35 cm×35 cm，每层扫描的心动周期时相数为20。扫描范围自心尖至心底覆盖整个RV和LV，共采集9层-13层。根据受试者心率不同，每层图像采集期间患者需屏气约8 s-14 s。

### 1.3 CMR图像分析与心功能参数计算

将CMR图像传输至AW4.3工作站（Advantage Windows version 4.3; GE Healthcare, Milwaukee, Wis）并通过Report Card 4.6软件进行图像观察和数据测量。心室形态和功能学参数测量方法如下：选择短轴位FIESTA序列图像，手动描记自心尖至心底各层面RV和LV的心外膜及心内膜轮廓，分别将心室容积达最大、最小的时相分别定义为舒张末期和收缩末期。心室容积包括其流出道容积，乳头肌和肌小梁计入心室腔内部分，其质量不计入心室质量。RV心肌质量为其游离壁心肌的质量，室间隔心肌的质量计入LV心肌质量。手动描记心室心外膜及心内膜轮廓后，软件可自动计算心室形态和功能学参数，并经体表面积（body surface area, BSA）校正后用于统计学分析，包括RV和LV的舒张末期容积指数（end-diastolic volume index, EDVI）、收缩末期容积指数（end-systolic volume index, ESVI）、每搏输出量指数（stroke volume index, SVI）、心指数（cardiac index, CI）、射血分数（ejection fraction, EF）、心肌质量指数（myocardial mass index, MMI）。体表面积（body surface area, BSA）估算公式为：BSA（m^2^）=0.006, 1×身高（cm）+0.012, 8×体重（kg）-0.152, 9。

### 1.4 统计学方法

采用SPSS 22.0统计软件进行统计数据分析。计量资料以均数±标准差（Mean±SD）来表示。采用单因素方差分析（*ANOVA*检验）比较三组间心功能参数的差别，事后多重比较用*LSD*检验。在RHC结果分析中，PH患者IVS无形变组和IVS有形变组的RHC参数，采用独立样本*t*检验。*P* < 0.05为差异有统计学意义。

## 2 结果

### 2.1 一般资料

36例PH患者，按照IVS有无形变分为两组：IVS无形变组（10例）（图 1B，图 1E）和IVS有形变组（26例）（图 1C，图 1F）。PH患者和健康志愿者（图 1A，图 1D）的一般资料见表 1。

**1:**
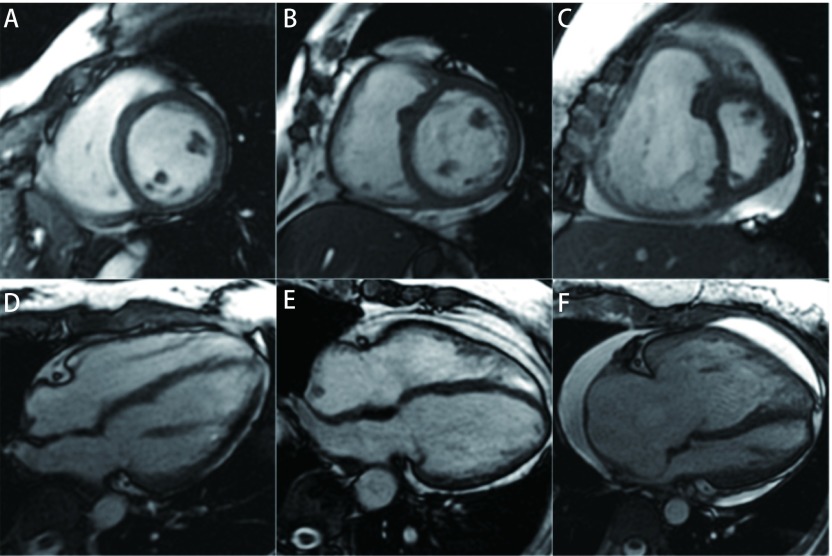
CMR图像，上行为短轴位，下行为四腔心位。A、D为正常对照组:女性，26岁；B、E为IVS无形变组：女性，65岁，mPAP=44 mmHg，IVS未发生形变；C、F为IVS有形变组：女性，31岁，mPAP=54 mmHg，RV明显增大，IVS形变并向左偏。 CMR imaging. Upper row is short axis image, and lower row is four-chamber image. Images A and D are in control group: female, 26 years old. Images B and E are in non-deformation group: female, 65 years old, mPAP=44 mmHg, IVS no deformation. Images C and F are in deformation group: female, 31 years old, mPAP=54 mmHg, RV enlarged significantly, IVS deformation.

**1:** 一般资料 Normal information

Parameters	Control group	IVS non-deformation group	IVS deformation group	*F*	*P*
Female (Total)	2 (22)	3 (10)	3 (26)		
Age (year)	40.9±9.2	43.9±12.6	45.6±16.9	1.452	0.360
HR (bpm)	68.6±10.8	71.9±13.5	86.8±10.9	21.854	< 0.001
BSA (kg/m^2^)	1.68±0.16	1.65±0.19	1.68±0.22	0.108	0.660
There was statistically significant among IVS deformation group, control group, and IVS non-deformation group, while no significant difference in the control group and IVS no-deformation group. HR: heart rate; BSA: body surface area.					

### 2.2 RHC结果

36例PH患者的RHC结果见表 2，IVS有形变组的平均肺动脉压（mean pulmonary arterial pressure, mPAP）、肺动脉收缩压（systolic pulmonary artery pressure, sPAP）、肺动脉舒张压（diatolic pulmonary artery pressure, dPAP）和肺血管阻力（pulmonary vascular resistance, PVR）均大于IVS无形变组。

**2:** RHC结果 The result of RHC

RHC parameters	IVS non-deformation group	IVS deformation group	*t*	*P*
mPAP (mmHg)	40.9±8.4	55.4±14.7	-2.925	0.006
sPAP (mmHg)	68.40±18.68	90.69±23.44	-2.689	0.011
dPAP (mmHg)	23.10±6.97	33.12±12.35	-2.408	0.022
PVR（Wood）	10.27±3.60	16.61±6.72	-2.815	0.001
mPAP: mean pulmonary arterial pressure; sPAP: systolic pulmonary artery pressure; dPAP: diatolic pulmonary artery pressure; PVR: pulmonary vascular resistance.				

### 2.3 心室功能参数的比较

IVS无形变组、IVS有形变组与对照组相比较，三组间心室功能参数比较见表 3。ANOVA分析三组整体结果显示，RVEDVI、RVESVI、RVSVI、RVCI、RVEF、RVMMI、LVEDVI、LVESVI、LVSVI、LVCI均存在统计学差异；而LVEF、LVMMI均无统计学差异。事后三组组间比较：（1）RV功能参数比较：IVS无形变组与对照组相比，RVEF显著减低，RVSVI减低。IVS有形变组与IVS无形变组相比，RVEDVI、RVESVI、RVCI、RVMMI均显著升高；而RVEF显著减低。IVS有形变组与对照组相比，RVEDVI、RVESVI、RVCI、RVMMI均显著升高；RVEF显著减低。（2）LV功能参数比较：IVS无形变组与对照组相比，LVEDVI、LVSVI减低。IVS有形变组与无形变组相比，LVEDVI、LVSVI显著减低，LVCI减低。IVS有形变组与对照组相比，LVEDVI、LVESVI、LVSVI、LVCI均显著减低。

**3:** IVS无形变组、IVS有形变组与对照组三组各心室功能参数 Ventricular function parameters of IVS non-deformation group, IVS deformation group and control group

Ventricular function parameters	Control group	IVS non-deformation group	IVS deformation group	*F*	*P*	*P1*	*P2*	*P3*
RV ventricular function parameters								
RVEDVI (mL/m^2^)	78.54±11.88	70.68±16.13	121.32±31.50	27.20	< 0.001	0.382	< 0.001	< 0.001
RVESVI (mL/m^2^)	35.97±7.38	40.73±8.14	83.68±21.10	65.59	< 0.001	0.417	< 0.001	< 0.001
RVSVI (mL/m^2^)	42.58±7.16	29.95±11.26	37.64±17.59	3.07	0.045	0.017	0.210	0.130
RVCI (L/m^2^)	2.89±0.46	2.11±0.93	4.83±3.29	7.36	0.001	0.369	0.004	0.002
RVEF (%)	54.34±5.85	41.45±9.38	30.56±10.24	44.85	< 0.001	< 0.001	< 0.001	0.001
RVMMI (g/m^2^)	15.74±9.78	14.79±2.67	47.06±1.64	5.74	0.005	0.944	0.003	0.017
LV ventricular function parameters								
LVEDVI (mL/m^2^)	83.44±15.90	70.31±25.43	50.43±13.88	22.72	< 0.001	0.048	< 0.001	0.003
LVESVI (mL/m^2^)	36.18±13.59	29.99±17.81	22.39±7.40	7.72	0.001	0.187	< 0.001	0.098
LVSVI (mL/m^2^)	47.41±6.33	40.29±9.05	28.04±7.68	41.10	< 0.001	0.015	< 0.001	< 0.001
LVCI (L/m^2^)	3.22±0.45	2.90±0.84	2.39±0.60	11.31	< 0.001	0.169	< 0.001	0.029
LVEF (%)	58.38±8.86	59.29±7.14	55.92±5.84	1.06	0.354	0.747	0.252	0.222
LVMMI (g/m^2^)	42.82±7.64	39.89±5.26	41.31±9.43	0.47	0.625	0.354	0.530	0.642
RVEDVI: right ventricle end-diastolic volume index; RVESVI: right ventricle end-systolic volume index; RVSVI: right ventricle stroke volume index; RVCI: right ventricle cardiac index; RVEF: right ventricle ejection fraction; RVMMI: right ventricle myocardial mass index; LVEDVI: left ventricle end-diastolic volume index; LVESVI: left ventricle end-systolic volume index; LVSVI: left ventricle stroke volume index; LVCI: left ventricle cardiac index; LVEF: left ventricle ejection fraction; LVMMI: left ventricle myocardial mass index. *P*: Statistical results among the three groups; *P1*: the control group compared with the IVS non-deformation group; *P2*: the control group compared with the IVS deformation group; *P3*: IVS no-deformation group compared with IVS deformation group.								

## 3 讨论

本研究中，IVS有形变组的肺动脉压力（mPAP、sPAP、dPAP）及肺循环阻力（PVR）增高程度均明显高于IVS无形变组。正常人的LV压力远高于RV压力，形成左心向右心的正性跨IVS压力梯度，IVS突向RV侧，短轴位示RV呈新月形，LV呈类圆形。PH患者的肺动脉压力增高，RV后负荷随之加重，正性跨IVS压力梯度逐渐减低，达到一定程度即可导致IVS向LV侧出现偏移，LV变形呈“D”形；当RV舒张压高于LV舒张压5 mmHg，则会出现IVS向左弓形突出（leftward ventricular septal bowing, LVSB），呈现出以右心为主导的状态^[4-10]^。由此可见，IVS形态的变化与两心室的压力变化密切相关，IVS的形态在一定程度上能够代表PH的严重程度。

### 3.1 PH患者IVS不同形态下RV功能特点

本研究结果表明，IVS尚未发生形变时，PH患者的RVEDV和RVESV亦无明显改变；然而，RVSV和RVEF已发生明显变化，且较正常组显著减低。RVSV和RVEF的减低反映了RV收缩功能受损，也提示了RV收缩功能受损出现在PH早期，可作为提示PH的早期指标。PH导致肺血管压力增高，肺循环阻力增加造成的RV后负荷增加，这是RVSV和RVEF降低的主要原因^[7, 11-12]^。

当IVS发生形变后，RVEDV、RVESV、RVMM均增高。RVEDV和RVESV的增高代表RV心腔的扩大，即RV心腔扩大不仅是RV游离壁外膨的结果，IVS的形变也起到一定作用。RVMM的增高则代表RV心肌质量的增加，反映了随着肺动脉压力的增高和病程的延长，RV心肌代偿性增厚的程度增加。本研究中的有形变组和无形变组PH患者的RVSV无差别，说明RV心腔扩大和心肌增厚的代偿性改变使得RV尚能维持其先前已经受损的SV，而且有形变组的RVCO高于无形变组，这可能是由于PH患者RV心肌壁增厚或心腔扩大等一系列变化和代偿机制允许RVSV增加来维持其心输出量^[13, 14]^。然而，IVS有形变组PH患者的RVEF低于无形变组，说明RV收缩功能受损程度进一步加重^[4, 15-17]^。

### 3.2 PH患者IVS不同形态下LV功能特点

IVS无形变组的LVEDV和LVSV明显低于正常对照组，说明PH早期即对LV功能产生了影响。其可能的机制是，RV收缩功能受损使肺循环血量减少，进而LV回心血量减少而影响其充盈，出现LVEDV及LVSV降低^[7, 11, 12]^。

IVS发生形变后LVEDV和LVSV进一步明显降低，而且LVCO也出现了明显减低，但其LVEF无明显下降，提示LV收缩功能未明显受损。PH患者肺循环压力明显增高，而对于体循环压力无直接影响，即对LV的压力负荷无直接影响^[14, 18]^。因此，LVEDV、LVSV和LVCO的降低主要由于LV容量负荷降低导致的。先前研究认为主要有两方面的机制：一方面是RV功能受损，造成的LV回心血量减少；另一方面是IVS的左移亦限制了LV在舒张早期的充盈^[3, 19]^。而IVS有形变组较无形变组的RVSV并未明显降低，甚至有增高趋势，又因IVS有形变组的心率增加，RVCO随之升高。这些参数的变化说明虽然IVS有形变组的RVEF进一步降低，收缩功能进一步受损，但相应的代偿机制能够维持RV的输出量，即维持LV的回心血量。本研究发现IVS有形变组PH患者LVEDV较无形变组降低，说明IVS形变限制了LV的充盈，造成LVEDV、LVSV和LVCO降低的主要原因可能是IVS左移限制了LV的充盈。

本研究存在一些不足之处：（1）本研究样本量相对较少、不平衡。就诊时PH大多已进展为中至重度，而IVS形态未发生形变者样本量较少。（2）CMR检查时间较长，需要配合屏气，不适用于心功能极差的PH病人。

综上，RVEF在IVS无形变时即受损，而LVEF并未明显受损，说明IVS无形变时RV收缩功能受损可能对全心功能的改变起显著作用；而IVS发生形变则提示RV收缩功能进一步受损，LV充盈受限，LV功能发生明显异常。IVS形变是易于观察且表现直观的形态学特征，通过观察IVS形变可推测PH患者心室功能的变化，对于临床制定PH患者干预方案有一定的参考价值。
